# Supplementary material for: The gut microbiome promotes mitochondrial respiration in the brain of a Parkinson’s disease mouse model
Source: NPJ Parkinsons Dis. 2025 Oct 20;11:301. doi: 10.1038/s41531-025-01142-5 (PMC12537952; doi:10.1038/s41531-025-01142-5)
Supplement: Supplementary file 1 — Morais_et_al_supplementary_materials_071725 [file 41531_2025_1142_MOESM1_ESM.pdf]

SUPPLEMENTARY INFORMATION

for

The gut microbiome promotes mitochondrial respiration in the brain of a Parkinson's disease mouse model

Livia H. Morais *et al.*

This file includes:

- Supplementary Figs. 1 through 8
- Supplementary Table 2

Other supplementary materials provided in separate files:

- Supplementary Table 1: Differentially-expressed genes between groups
  Estimated log_2 fold changes and p-values for genes differentially expressed in various group comparisons.
- Source data for Fig. 3a-f

FIGURE S1

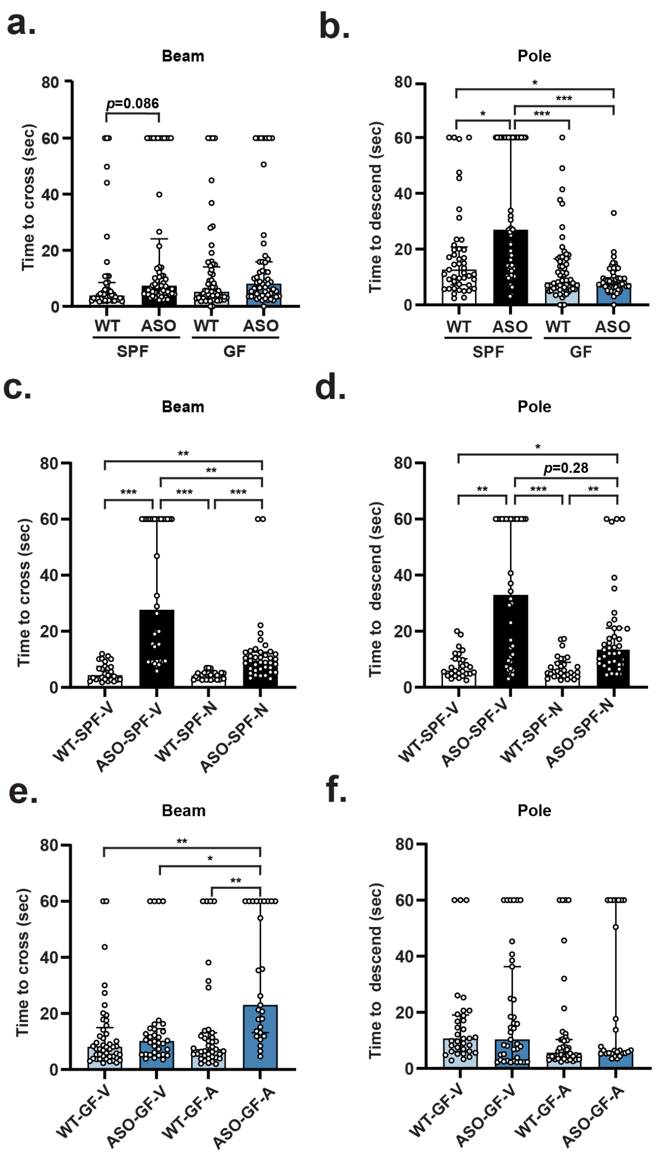
a.
Beam
p=0.086
Time to cross (sec)
WT ASO WT ASO
SPF GF
b.
Pole
Time to descend (sec)
WT ASO WT ASO
SPF GF
c.
Beam
Time to cross (sec)
WT-SPF-V
ASO-SPF-V
WT-SPF-N
ASO-SPF-N
d.
Pole
p=0.28
Time to descend (sec)
WT-SPF-V
ASO-SPF-V
WT-SPF-N
ASO-SPF-N
e.
Beam
Time to cross (sec)
WT-GF-V
ASO-GF-V
WT-GF-A
ASO-GF-A
f.
Pole
Time to descend (sec)
WT-GF-V
ASO-GF-V
WT-GF-A
ASO-GF-A

Supplementary Fig. 1. Motor behavior plots displaying all trial values.

(a–b) Bar plots represent the time taken to cross the beam or descend the pole, with individual trials plotted for comparisons between specific pathogen free (SPF) and germ-free (GF) mice. **(c–d)** Bar plots represent the time taken to cross the beam or descend the pole, with individual trials plotted for comparisons between ASO-SPF mice with NAC treatment (N) and vehicle treatment (V). **(e–f)** Bar plots represent the time taken to cross the beam or descend the pole, with individual trials plotted for comparisons between ASO-GF mice with and without Auranofin treatment (A) and vehicle treatment (V). SPF, specific pathogen-free; GF, germ-free; WT, wild-type; ASO, Thy1-α-synuclein overexpressing; NAC, N-acetyl-L-cysteine (N); V, vehicle; A, Auranofin. Sample size: n = 9–19 mice per group; 1–3 trials per mouse. Each dot represents a single trial.

Statistical details: (**a-f**) Bar plots represent the time taken to cross the beam or descend the pole, with individual trials plotted for comparisons between groups. Data are shown as median with interquartile range. Pairwise comparisons were calculated on median values with Kruskal-Wallis test followed by Conover's post-hoc test. Significance: * $p < 0.05$, ** $p < 0.01$, *** $p < 0.001$. Sample size: n = 9–19 mice per group; 1–3 trials per mouse.

FIGURE S2

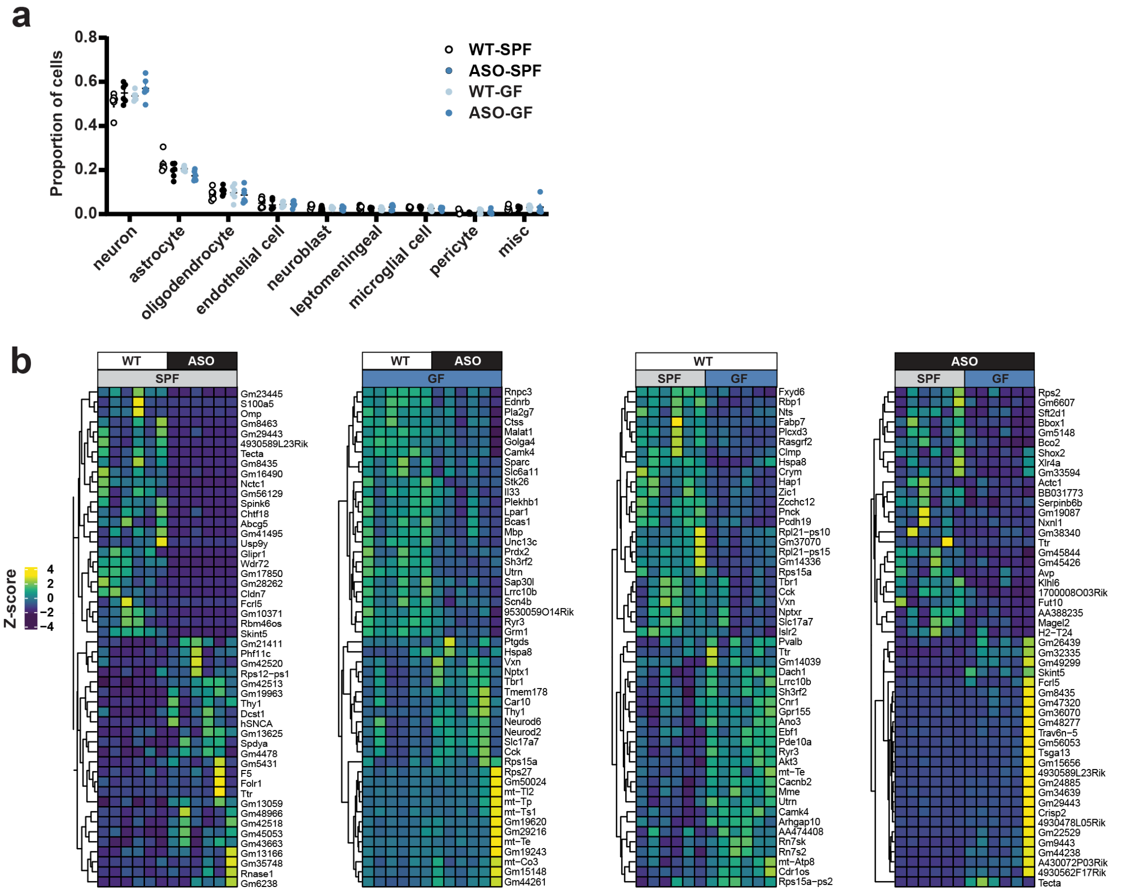

Supplementary Fig. 2. No differences in cell-type enrichment across animal groups.

Bulk quantitative RNA-sequencing of whole striatum tissue. Differential gene expression between groups was assessed using the DESeq2 package comparing the effects of genotype (WT x ASO) and the gut microbiome (SPF x GF). (**a**) Cell-type enrichment analysis of all differentially expressed genes (DEGs) detected no differences between groups. (**b**) Heat map showing the top 25 up- and downregulated genes in each comparison. SPF, specific pathogen-free; GF, germ-free; WT, wild-type; ASO, Thy1-α-synuclein overexpressing.

FIGURE S3

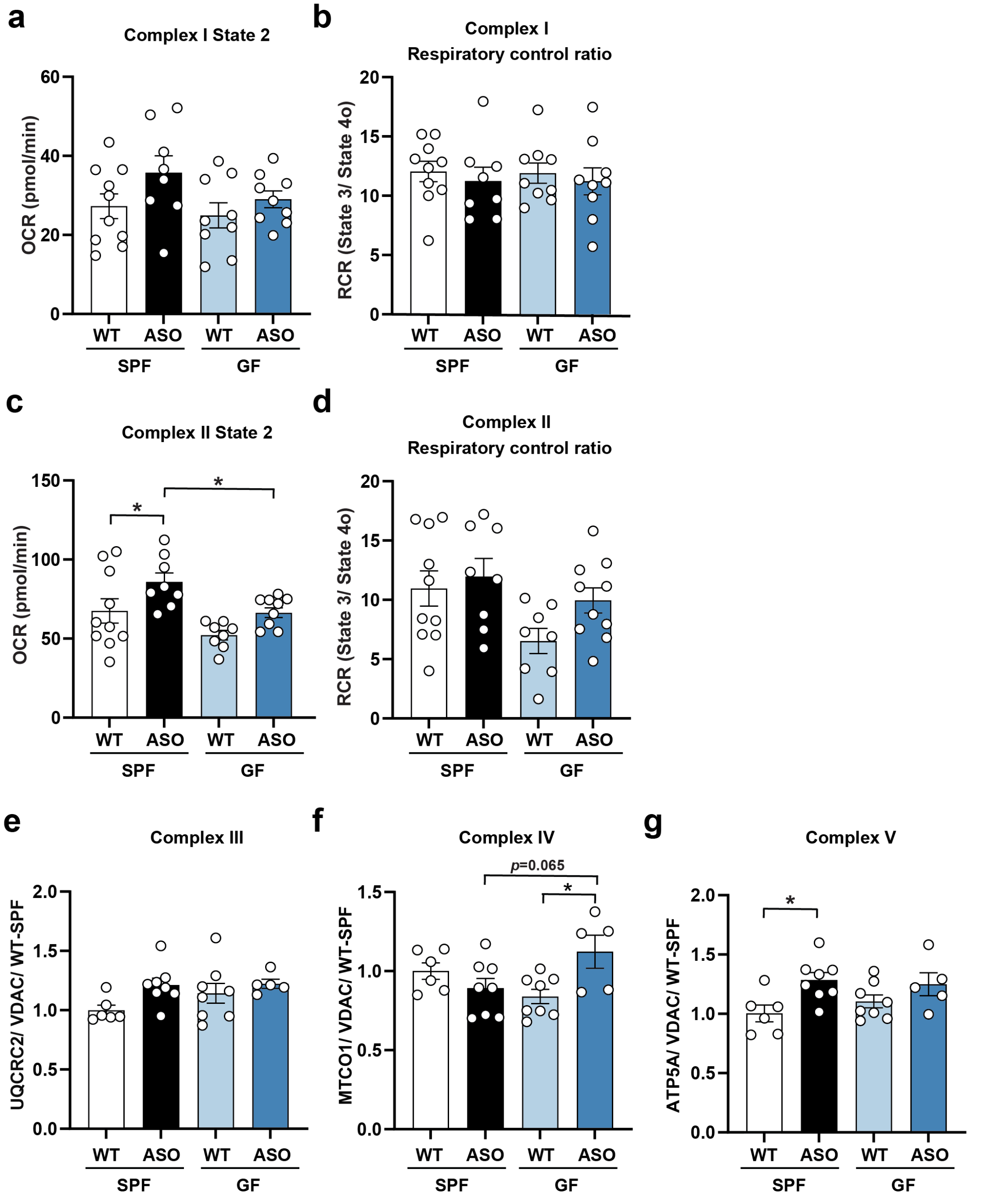
a
Complex I State 2
OCR (pmol/min)
60
40
20
0
WT
ASO
WT
ASO
SPF
GF
b
Complex I
Respiratory control ratio
RCR (State 3/ State 4o)
20
15
10
5
0
c
Complex II State 2
OCR (pmol/min)
150
100
50
0
*
*
d
Complex II
Respiratory control ratio
RCR (State 3/ State 4o)
e
Complex III
UQCRC2/ VDAC/ WT-SPF
2.0
1.5
1.0
0.5
0.0
f
Complex IV
MTCO1/ VDAC/ WT-SPF
p=0.065
*
g
Complex V
ATP5A/ VDAC/ WT-SPF
*

Supplementary Fig. 3. The gut microbiome regulates mitochondrial respiration in Thy1-ASO mice.

(**a-d**) Oxygen consumption ratio (OCR) was measured in freshly isolated striatal mitochondria using Seahorse, evaluating Complex I and Complex II respiration. (**a, c**) State 2. (**b, d**) Respiratory control ratio (RCR). (**e-g**) Quantification of levels of oxidative phosphorylation (OxPhos) complexes, normalized to voltage-dependent anion channel 1 (VDAC1), in isolated mitochondria by Western blotting. (**e**) Complex III UQCRC2 subunit. (**f**) Complex IV MTCO1 subunit. (**g**) Complex V ATP5A subunit. SPF, specific pathogen-free; GF, germ-free; WT, wild-type; ASO, Thy1-α-synuclein overexpressing; RCR, respiratory control ratio; CI, Complex I; CII, Complex II; CIII, Complex III; CIV, Complex IV; CV, Complex V; UQCRC2, ubiquinol-cytochrome c reductase core protein 2; MTCO1, cytochrome c oxidase subunit 1; ATP5A, ATP synthase subunit alpha.

Statistical details: (**a-g**) Data were analyzed using a linear model (variable ~ Genotype + Microbiome + Genotype*Microbiome) and pairwise comparisons with Benjamini-Hochberg (FDR) correction. Data are expressed as mean ± SEM. Significance: * $p < 0.05$. (**a-d**) Sample size: n=8-10 per group, data combined from 4 different cohorts. (**e-g**) Sample size: n=6-8 per group, data combined from 2 different cohorts.

FIGURE S4

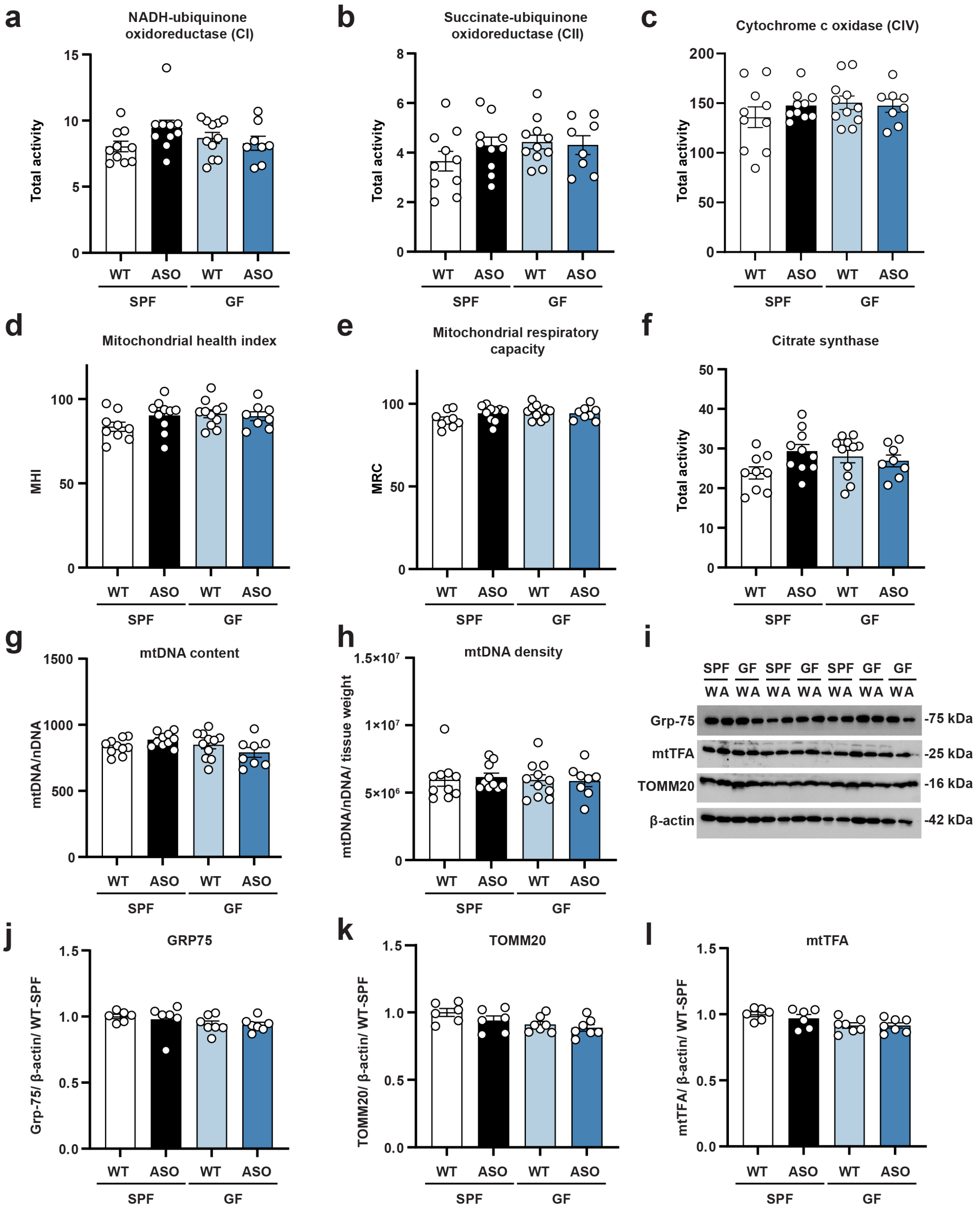
a
NADH-ubiquinone oxidoreductase (CI)
Total activity
b
Succinate-ubiquinone oxidoreductase (CII)
Total activity
c
Cytochrome c oxidase (CIV)
Total activity
d
Mitochondrial health index
MHI
e
Mitochondrial respiratory capacity
MRC
f
Citrate synthase
Total activity
g
mtDNA content
mtDNA/nDNA
h
mtDNA density
mtDNA/nDNA/ tissue weight
i
SPF GF SPF GF SPF GF GF
W A W A W A W A W A W A W A
Grp-75
mtTFA
TOMM20
β-actin
-75 kDa
-25 kDa
-16 kDa
-42 kDa
j
GRP75
Grp-75/ β-actin/ WT-SPF
k
TOMM20
TOMM20/ β-actin/ WT-SPF
l
mtTFA
mtTFA/ β-actin/ WT-SPF
WT ASO WT ASO
SPF GF

Supplementary Fig. 4. Mitochondrial abundance is not altered by the gut microbiome or by genotype. (**a-c**) Mitochondrial enzymatic activity in total striatum tissue extracts for (**a**) NADH-ubiquinone oxidoreductase (CI), (**b**) succinate-ubiquinone oxidoreductase (CII), (**c**) cytochrome c oxidase (CIV). (**d**) Mitochondrial health index (MHI), calculated using the equation MHI = (CI + CII + CIV) / (CS + mtDNA density + 1)×100. (**e**) Mitochondrial respiratory capacity (MRC), calculated using the equation MRC = (Average √CI, √CII, √CIV) / (Average 3√CS, 3√mtDNA density)×100. (**f-h**) Mitochondrial content in total striatum extracts. (**f**) Citrate synthase activity. (**g)** mtDNA copy number measured by qPCR, subtracting mtDNA Ct from average nDNA Ct, calculated by 2^(ΔCt) x 2. (**h**) mtDNA density, linearizing Ct as 2^Ct / (1/10^(-12)) to derive mtDNA abundance per unit of tissue. (**i-l**) Western blotting for mitochondrial markers in total striatum protein extracts. (**i**) Image of representative blot. See Supplementary Figs. 7 and 8 for uncropped images of blots. Relative levels of (**j**) GRP75, (**k**) TOMM20, and (**l**) mtTFA in different groups. Protein levels were normalized to β-actin. SPF, specific pathogen-free; GF, germ-free; WT/W, wild-type; ASO/A, Thy1-α-synuclein overexpressing; CI, Complex I; CII, Complex II; CIV, Complex IV; CS, citrate synthase; Ct, cycle threshold; GRP75, glucose-regulated protein 75; TOMM20, translocase of outer mitochondrial membrane; mtTFA, mitochondrial transcription factor A.

Statistical details: (**a-h**) Data were analyzed using a linear model (variable ~ Genotype + Microbiome + Genotype*Microbiome*)* and pairwise comparisons with Benjamini-Hochberg (FDR) correction. Data are expressed as mean ± SEM. Sample size: n=6-7, data combined from 2 different cohorts. (**j-l**) Data were analyzed using a linear model (variable ~ Genotype + Microbiome + Genotype*Microbiome) and pairwise comparisons with Benjamini-Hochberg (FDR) correction. Data are expressed as mean ± SEM. Sample size: n=9-11, data combined from 2 different cohorts.

FIGURE S5

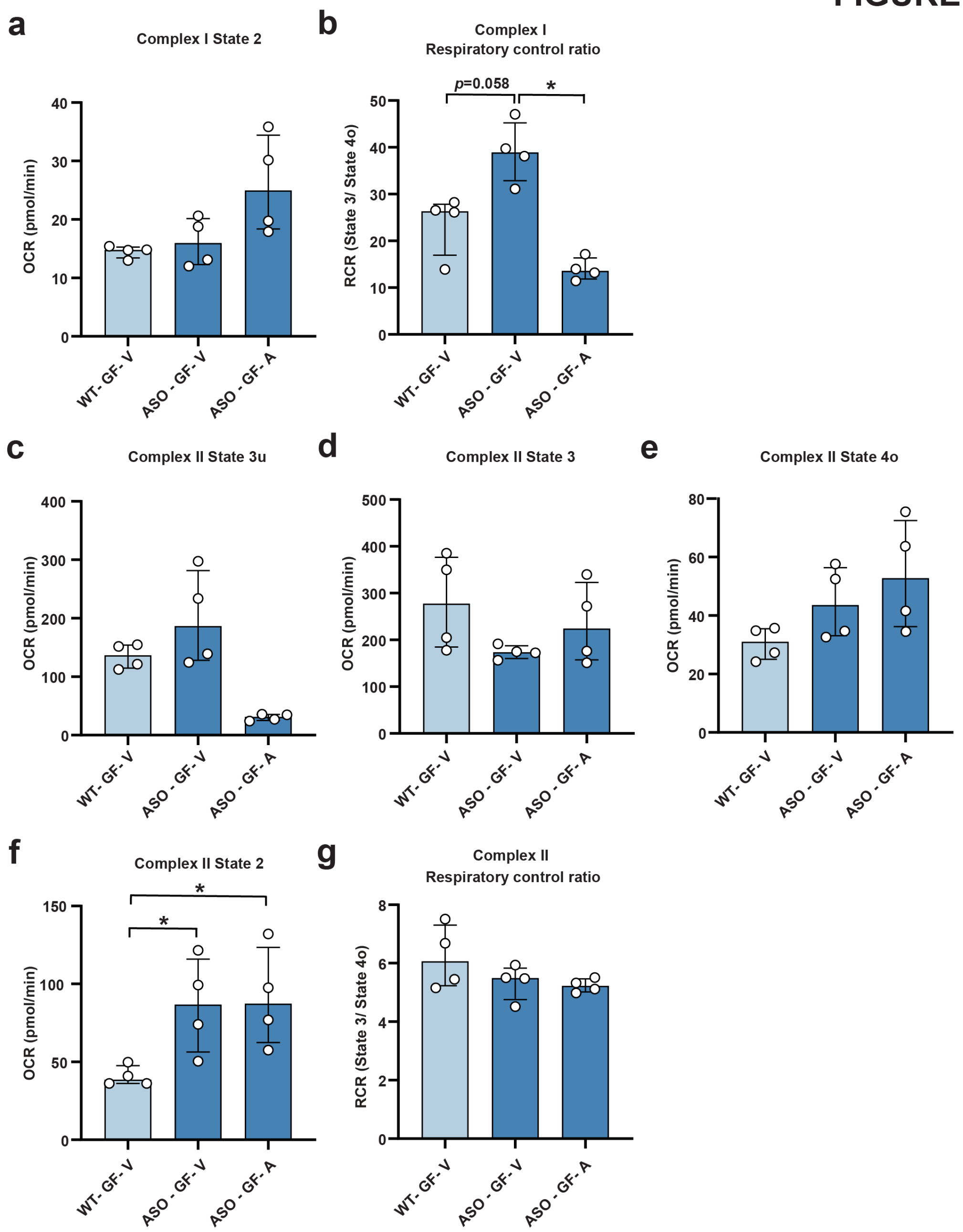
a
Complex I State 2
OCR (pmol/min)
0
10
20
30
40
WT- GF- V
ASO - GF- V
ASO - GF- A
b
Complex I
Respiratory control ratio
RCR (State 3/ State 4o)
p=0.058
*
0
10
20
30
40
50
c
Complex II State 3u
OCR (pmol/min)
0
100
200
300
400
d
Complex II State 3
OCR (pmol/min)
0
100
200
300
400
500
e
Complex II State 4o
OCR (pmol/min)
0
20
40
60
80
f
Complex II State 2
OCR (pmol/min)
*
*
0
50
100
150
g
Complex II
Respiratory control ratio
RCR (State 3/ State 4o)
0
2
4
6
8

Supplementary Fig. 5. TXNRD inhibition does not affect Complex II activity in ASO-GF mice.

(**a-g**) Oxygen consumption rate (OCR) was measured in freshly isolated mitochondria using Seahorse, evaluating Complex I (CI) and Complex II (CII) respiration. (**a**) CI State 2. (**b**) CI respiratory control ratio (RCR). (**c**) CII State 3u (uncoupled, maximal respiration). (**d**) CII State 3 (ATP-linked respiration). (**e**) CII State 4o (proton leak). (**f**) CII State 2. (**g**) CII RCR. SPF, specific pathogen-free; GF, germ-free; WT, wild-type; ASO, Thy1-α-synuclein overexpressing; V, vehicle; A, Auranofin.

Statistical details: (**a-g**) Data were analyzed by Kruskal-Wallis test followed by Dunn's post-hoc test with Benjamini-Hochberg (FDR) correction. Significance: * $p < 0.05$. Data are expressed as median ± interquartile range. Data combined from 2 different cohorts. Sample size: n=4 per group.

FIGURE S6

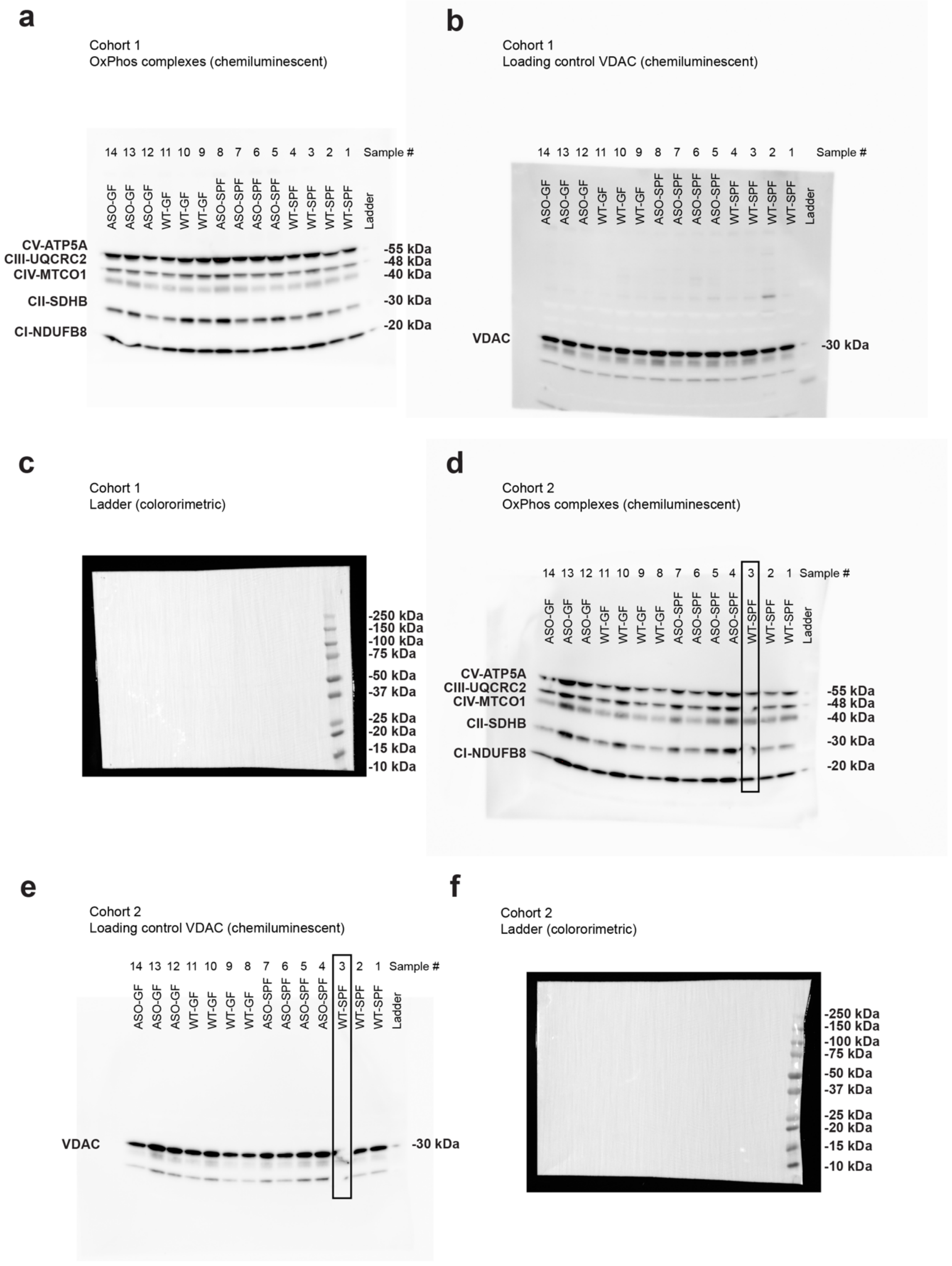

a
Cohort 1
OxPhos complexes (chemiluminescent)
14 13 12 11 10 9 8 7 6 5 4 3 2 1 Sample #
ASO-GF ASO-GF ASO-GF WT-GF WT-GF WT-GF ASO-SPF ASO-SPF ASO-SPF ASO-SPF WT-SPF WT-SPF WT-SPF WT-SPF Ladder
CV-ATP5A
CIII-UQCRC2
CIV-MTCO1
CII-SDHB
CI-NDUFB8
-55 kDa
-48 kDa
-40 kDa
-30 kDa
-20 kDa
b
Cohort 1
Loading control VDAC (chemiluminescent)
14 13 12 11 10 9 8 7 6 5 4 3 2 1 Sample #
ASO-GF ASO-GF ASO-GF WT-GF WT-GF WT-GF ASO-SPF ASO-SPF ASO-SPF ASO-SPF WT-SPF WT-SPF WT-SPF WT-SPF Ladder
VDAC
-30 kDa
c
Cohort 1
Ladder (colororimetric)
-250 kDa
-150 kDa
-100 kDa
-75 kDa
-50 kDa
-37 kDa
-25 kDa
-20 kDa
-15 kDa
-10 kDa
d
Cohort 2
OxPhos complexes (chemiluminescent)
14 13 12 11 10 9 8 7 6 5 4 3 2 1 Sample #
ASO-GF ASO-GF ASO-GF WT-GF WT-GF WT-GF WT-GF ASO-SPF ASO-SPF ASO-SPF ASO-SPF WT-SPF WT-SPF WT-SPF Ladder
CV-ATP5A
CIII-UQCRC2
CIV-MTCO1
CII-SDHB
CI-NDUFB8
-55 kDa
-48 kDa
-40 kDa
-30 kDa
-20 kDa
e
Cohort 2
Loading control VDAC (chemiluminescent)
14 13 12 11 10 9 8 7 6 5 4 3 2 1 Sample #
ASO-GF ASO-GF ASO-GF WT-GF WT-GF WT-GF WT-GF ASO-SPF ASO-SPF ASO-SPF ASO-SPF WT-SPF WT-SPF WT-SPF Ladder
VDAC
-30 kDa
f
Cohort 2
Ladder (colororimetric)
-250 kDa
-150 kDa
-100 kDa
-75 kDa
-50 kDa
-37 kDa
-25 kDa
-20 kDa
-15 kDa
-10 kDa

Supplementary Fig. 6. Uncropped images of Western blots for oxidative phosphorylation (OxPhos) complexes in isolated mitochondria (related to Figure 2g).

(a) OxPhos complex subunits, **(b)** loading control (VDAC), and **(c)** molecular weight ladder for experimental cohort 1. **(d-f)** Cohort 2. Proteins of interest were visualized using chemiluminescent detection; molecular weight ladder was imaged using a colorimetric setting. Images were acquired with a ChemiDoc imaging system. Boxes indicate a sample excluded due to technical error (Cohort 2, Sample #3). SPF, specific pathogen-free; GF, germ-free; WT, wild-type; Thy1-ASO, Thy1-α-synuclein overexpressing; CI–CV, mitochondrial respiratory complexes I–V; NDUFB8, NADH dehydrogenase [ubiquinone] 1 beta subcomplex subunit 8; SDHB, succinate dehydrogenase subunit B; UQCRC2, ubiquinol-cytochrome c reductase core protein 2; MTCO1, cytochrome c oxidase subunit 1; ATP5A, ATP synthase subunit alpha.

FIGURE S7

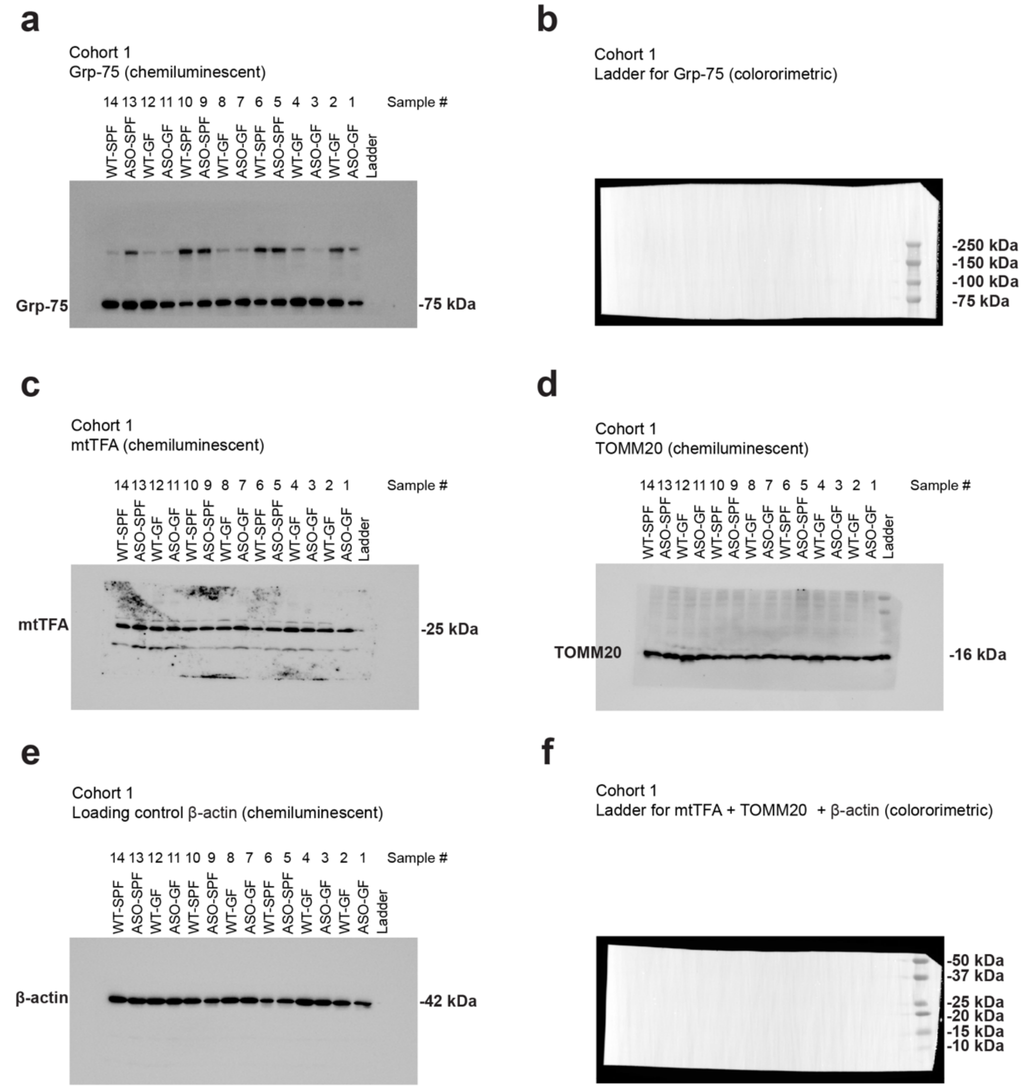

Supplementary Fig. 7. Uncropped images of Western blots for mitochondrial markers in total striatum protein extracts from Cohort 1 (related to Supplementary Fig. 4i).

(a) GRP75 and **(b)** associated molecular weight ladder. **(c)** mtTFA, **(d)** TOMM20, **(e)** loading control (β-actin), and **(f)** associated molecular weight ladder. Proteins of interest were detected using chemiluminescent detection; molecular weight ladder was imaged using a colorimetric setting. Images were acquired with a ChemiDoc imaging system. The membrane was cut after transfer and before antibody probing. SPF, specific pathogen-free; GF, germ-free; WT, wild-type; Thy1-ASO, Thy1-α-synuclein overexpressing; GRP75, glucose-regulated protein 75; TOMM20, translocase of the outer mitochondrial membrane 20; mtTFA, mitochondrial transcription factor A.

FIGURE S8

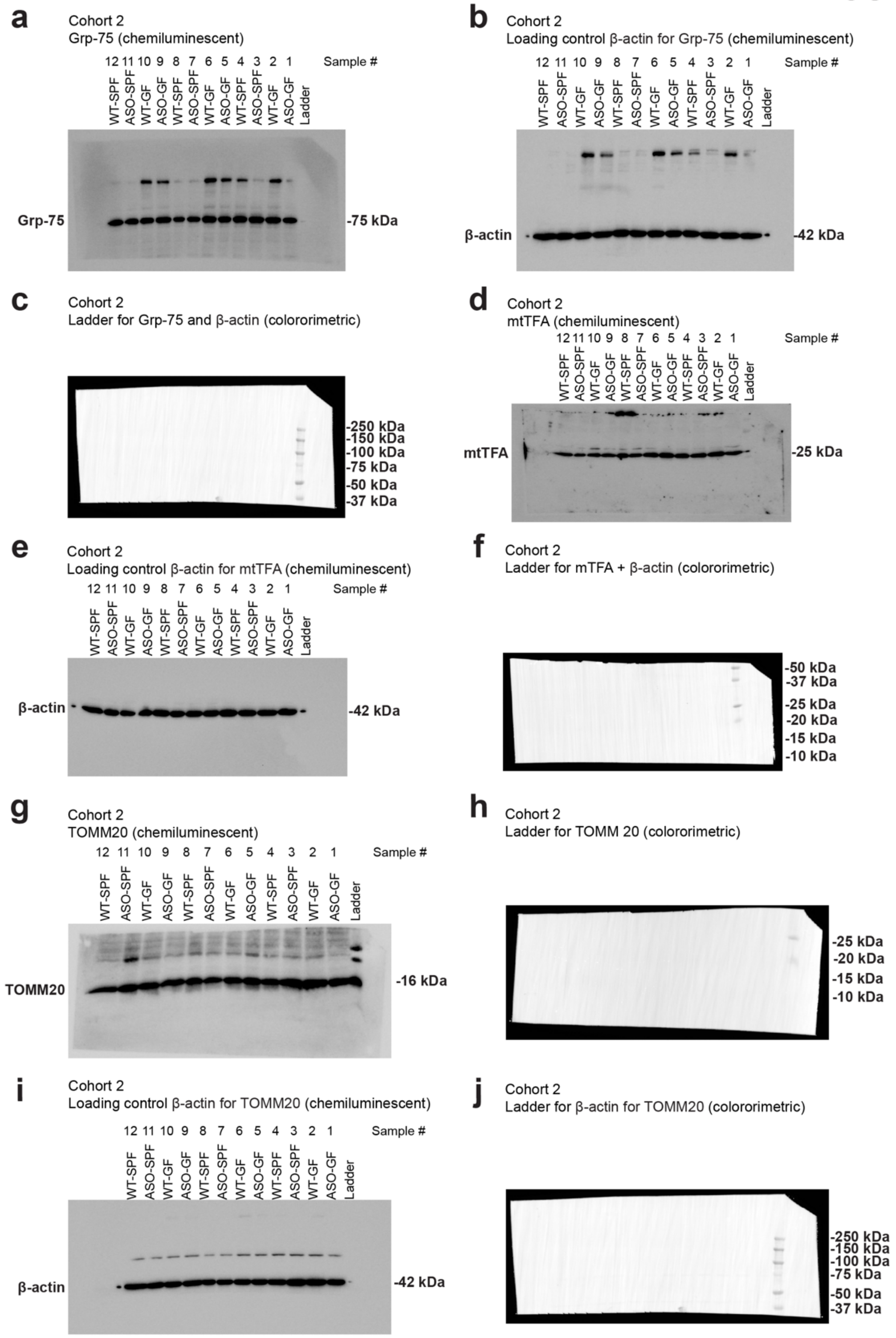
a
Cohort 2
Grp-75 (chemiluminescent)
12 11 10 9 8 7 6 5 4 3 2 1 Sample #
WT-SPF ASO-SPF WT-GF ASO-GF WT-SPF ASO-SPF WT-GF ASO-GF WT-SPF ASO-SPF WT-GF ASO-GF Ladder
Grp-75
-75 kDa
b
Cohort 2
Loading control β-actin for Grp-75 (chemiluminescent)
12 11 10 9 8 7 6 5 4 3 2 1 Sample #
WT-SPF ASO-SPF WT-GF ASO-GF WT-SPF ASO-SPF WT-GF ASO-GF WT-SPF ASO-SPF WT-GF ASO-GF Ladder
β-actin
-42 kDa
c
Cohort 2
Ladder for Grp-75 and β-actin (colororimetric)
-250 kDa
-150 kDa
-100 kDa
-75 kDa
-50 kDa
-37 kDa
d
Cohort 2
mtTFA (chemiluminescent)
12 11 10 9 8 7 6 5 4 3 2 1 Sample #
WT-SPF ASO-SPF WT-GF ASO-GF WT-SPF ASO-SPF WT-GF ASO-GF WT-SPF ASO-SPF WT-GF ASO-GF Ladder
mtTFA
-25 kDa
e
Cohort 2
Loading control β-actin for mtTFA (chemiluminescent)
12 11 10 9 8 7 6 5 4 3 2 1 Sample #
WT-SPF ASO-SPF WT-GF ASO-GF WT-SPF ASO-SPF WT-GF ASO-GF WT-SPF ASO-SPF WT-GF ASO-GF Ladder
β-actin
-42 kDa
f
Cohort 2
Ladder for mTFA + β-actin (colororimetric)
-50 kDa
-37 kDa
-25 kDa
-20 kDa
-15 kDa
-10 kDa
g
Cohort 2
TOMM20 (chemiluminescent)
12 11 10 9 8 7 6 5 4 3 2 1 Sample #
WT-SPF ASO-SPF WT-GF ASO-GF WT-SPF ASO-SPF WT-GF ASO-GF WT-SPF ASO-SPF WT-GF ASO-GF Ladder
TOMM20
-16 kDa
h
Cohort 2
Ladder for TOMM 20 (colororimetric)
-25 kDa
-20 kDa
-15 kDa
-10 kDa
i
Cohort 2
Loading control β-actin for TOMM20 (chemiluminescent)
12 11 10 9 8 7 6 5 4 3 2 1 Sample #
WT-SPF ASO-SPF WT-GF ASO-GF WT-SPF ASO-SPF WT-GF ASO-GF WT-SPF ASO-SPF WT-GF ASO-GF Ladder
β-actin
-42 kDa
j
Cohort 2
Ladder for β-actin for TOMM20 (colororimetric)
-250 kDa
-150 kDa
-100 kDa
-75 kDa
-50 kDa
-37 kDa

Supplementary Fig. 8. Uncropped images of Western blots for mitochondrial markers in total striatum protein extracts from Cohort 2 (related to Supplementary Fig. 4i).

(a) GRP75, **(b)** loading control (β-actin), and **(c)** associated molecular weight ladder. **(d)** mtTFA, **(e)** loading control (β-actin), and **(f)** associated molecular weight ladder. **(g)** TOMM20 and **(h)** associated molecular weight ladder. **(i)** Loading control (β-actin) and **(j)** associated molecular weight ladder. Proteins of interest were detected using chemiluminescent detection; molecular weight ladder was imaged using a colorimetric setting. Images were acquired with a ChemiDoc imaging system. The membranes were cut after transfer and before antibody probing. SPF, specific pathogen-free; GF, germ-free; WT, wild-type; Thy1-ASO, Thy1-α-synuclein overexpressing; GRP75, glucose-regulated protein 75; TOMM20, translocase of the outer mitochondrial membrane 20; mtTFA, mitochondrial transcription factor A.

Supplementary Table 2. Key Resource Table
Data, code, protocols, and key lab materials used in this study.

RESOURCE TYPE	RESOURCE NAME	SOURCE	IDENTIFIER	NEW/ REUSE	ADDITIONAL INFORMATION
Dataset	Tabular behavior data	GitHub	https://doi.org/10.5281/zenodo.14560061	new	data from pole descent and beam traversal assays shown in Figures 1A-B and 4A-D
Dataset	Tabular mitochondrial function data	GitHub	https://doi.org/10.5281/zenodo.14560061	new	data from mitochondrial function profiling shown in Fig. 2, Fig. 4E-G, S2, S3, and S4
Dataset	Transcriptomics data	GitHub	https://doi.org/10.5281/zenodo.14560061	new	data from transcriptomics shown in Fig. 1C-G and S1
Dataset	Proteomics data	GitHub	https://doi.org/10.5281/zenodo.14560061	new	data from proteomics shown in Fig. 3A-F
Software/code	Analysis and visualization code	GitHub	https://doi.org/10.5281/zenodo.14560061	new	code used to analyze all data
Software/code	Rsubread v2.16.1	https://bioconductor.org/packages/release/bioc/html/Rsubread.html	RRID:SCR_016945	reuse	
Software/code	limma v3.60.4	https://bioconductor.org/packages/release/bioc/html/limma.html	RRID:SCR_010943	reuse	
Software/code	DESeq2 v1.42.1	https://bioconductor.org/packages/release/bioc/html/DESeq2.html	RRID:SCR_015687	reuse	
Software/code	RITAN v1.26.0	https://bioconductor.org/packages/release/bioc/html/RITAN.html	N/A	reuse	
Software/code	Xcalibur	https://www.thermofisher.com/order/catalog/product/OPTON-30965	RRID:SCR_014593	reuse	
Software/code	Proteome Discoverer v2.5	https://www.thermofisher.com/order/catalog/product/IQLAAEGABSFAKJMAUH	RRID:SCR_014477	reuse	
Software/code	UniProtKB	http://www.uniprot.org/help/uniprotkb	RRID:SCR_004426	reuse	
Software/code	Sequest with Percolator	http://noble.gs.washington.edu/proj/percolator/	N/A	reuse	
Software/code	MitoCarta v3.0	http://www.broadinstitute.org/pubs/MitoCarta/	RRID:SCR_018165	reuse	
Software/code	Python v3.11.5	http://www.python.org	RRID:SCR_008394	reuse	
Software/code	NumPy v1.24.3	http://www.numpy.org	RRID:SCR_008633	reuse	
Software/code	Pandas v2.1.1	https://pandas.pydata.org	RRID:SCR_018214	reuse	
Software/code	Numba v0.58.0	https://numba.readthedocs.io/en/stable/index.html	RRID_SCR_025056	reuse	
Software/code	Bokeh v3.2.0	https://bokeh.org	N/A	reuse	

Software/code	bebi103 v0.1.17	https://bebi103.github.io	N/A	reuse	
Software/code	JupyterLab v4.0.6	http://jupyterlab.github.io/jupyterlab/	RRID:SCR_023339	reuse	
Software/code	R v4.3.2	https://www.r-project.org/	RRID:SCR_001905	reuse	
Protocol	Pole descent	protocols.io	dx.doi.org/10.17504/protocols.io.n92ld8j87v5b/v1	reuse	
Protocol	Beam traversal	protocols.io	dx.doi.org/10.17504/protocols.io.e6nvw1212lmk/v1	new	
Protocol	Tissue dissection	protocols.io	dx.doi.org/10.17504/protocols.io.x54v92z2ql3e/v1	new	
Protocol	Oxidative stress quantification	protocols.io	dx.doi.org/10.17504/protocols.io.4r3l2qbqjl1y/v1	new	
Protocol	Mitochondrial isolation	protocols.io	dx.doi.org/10.17504/protocols.io.5jyl82qz6l2w/v1	new	
Protocol	Mitochondrial respirometry	protocols.io	dx.doi.org/10.17504/protocols.io.bp2l62z4zgqe/v1	new	
Protocol	Western blotting	protocols.io	dx.doi.org/10.17504/protocols.io.x54v92z2ql3e/v1	new	
Protocol	Immuno-histochemistry	protocols.io	dx.doi.org/10.17504/protocols.io.kqdg32y61v25/v1	new	
Protocol	Mitochondrial proteomics	protocols.io	dx.doi.org/10.17504/protocols.io.36wgqny15gk5/v1	new	
Antibody	Rabbit anti-TFAM	ABclonal	Cat# A1926, RRID:AB_2763953	reuse	1:500 dilution
Antibody	Donkey anti-Rabbit IgG H&L Alexa Fluor 647	Abcam	Cat# ab150075, RRID:AB_2752244	reuse	1:500 dilution
Chemical, peptide, or recombinant protein	N-acetyl-L-cysteine	Sigma-Aldrich	Cat. #A7250	reuse	
Chemical, peptide, or recombinant protein	Auranofin	Cayman	Cat. #15316	reuse	
Critical commercial assay	OxiSelect In Vitro ROS/RNS Assay Kit	CellBio Labs	Cat. #STA-347-5	reuse	
Experimental model: Organism/strain	"Line 61" Thy1-αSyn mice	Masliah Lab	N/A	reuse	Chesselet et al. Neurother 9, 297–314 (2012); Rockenstein et al J Neurosci Res 68:568–578 (2002)
Experimental model: Organism/strain	BDF1 mice	Charles River	RRID:IMSR_CRL:099	reuse	

Oligonucleotide	COX1-Fwd	Rosenberg et al. Nat Commun 14, 4726 (2023)	N/A	reuse	Sequence: ACCACCATCATTTCTCCTTCTC
Oligonucleotide	COX1-Rev	Rosenberg et al. Nat Commun 14, 4726 (2023)	N/A	reuse	Sequence: CTCCTGCATGGGCTAGATTT
Oligonucleotide	COX1-Probe	Rosenberg et al. Nat Commun 14, 4726 (2023)	N/A	reuse	Sequence: HEX/AAGCAGGAG/ZEN/CAGGAACAGGATGAA/3IABkFQ
Oligonucleotide	B2M-Fwd	Rosenberg et al. Nat Commun 14, 4726 (2023)	N/A	reuse	Sequence: GAGAATGGGAAGCCGAACATA
Oligonucleotide	B2M-Rev	Rosenberg et al. Nat Commun 14, 4726 (2023)	N/A	reuse	Sequence: CCGTTCTTCAGCATTTGGATTT
Oligonucleotide	B2M-Probe	Rosenberg et al. Nat Commun 14, 4726 (2023)	N/A	reuse	Sequence: FAM/CGTAACACA/ZEN/GTTCCACCCGCCTC/3IABkFQ
